# Acute bi-ventricular haemodynamic changes after transcatheter aortic valve implantation for severe aortic stenosis assessed using invasive pressure-volume loops

**DOI:** 10.1007/s12928-026-01295-x

**Published:** 2026-05-23

**Authors:** Vitaliy Androshchuk, Edouard Long, Omar Chehab, Joshua Wilcox, Benedict McDonaugh, Ronak Rajani, Bernard Prendergast, Tiffany Patterson, Simon Redwood

**Affiliations:** 1 School of Cardiovascular Medicine & Sciences, Faculty of Life Sciences & Medicine, King’s College London, London, UK; 2 Cardiovascular Directorate, St Thomas’ Hospital, London, UK; 3 School of Biomedical Engineering and Imaging Sciences, Faculty of Life Sciences & Medicine, King’s College London, London, UK; 4 Cleveland Clinic London, London, UK; 5 King’s College London, The Reyne Institute, London, SE1 7EH UK

**Keywords:** Aortic stenosis, Left ventricle, Pressure-volume loops, Right ventricle, Transcatheter Aortic valve implantation.

## Abstract

Transcatheter aortic valve implantation (TAVI) aims to relieve the increased left ventricular (LV) afterload imposed by aortic stenosis (AS). However, the early haemodynamic impact of TAVI on bi-ventricular performance remains poorly characterised. This prospective study quantified the acute bi-ventricular response following TAVI for severe symptomatic AS using gold-standard invasive pressure-volume loop (PVL) assessment. Left and right ventricular (RV) PVLs were recorded using a conductance catheter pre- and immediately post-TAVI. Indices of contractile function, ventriculo-arterial coupling and energetics were derived. In total, 12 patients (84.1 years [interquartile range: 77.1, 87.2]; female 25%) were included. TAVI resulted in significant bi-ventricular reductions in afterload, measured by effective arterial elastance (LV: 2.1 [1.8, 2.3] to 1.4 [1.3, 1.7] mmHg/mL, *p* < 0.001; RV: 0.5 [0.4, 0.6] to 0.4 [0.3, 0.4] mmHg/mL, *p* = 0.006). Despite an acute decline in LV contractility, measured by end-systolic elastance (*p* = 0.021), there was a significant net improvement in ventriculo-arterial coupling in both the LV (*p* = 0.003) and RV (*p* = 0.006). Both the LV and RV demonstrated significant reductions in stroke work (SW) (LV-SW: 11,915.0 [9,727.5, 15,288.7] to 7,360.5 [6,937.0, 9,113.1] mmHg/mL, *p* < 0.001; RV-SW: 1,506.9 [1,420.5, 1,676.2] to 1,418.1 [1,250.8, 1,510.3] mmHg/mL, *p* = 0.020) and pressure volume area (PVA) (LV-PVA: 16,188.5 [13,171.5, 18,726.5] to 9,674.2 [9,185.9, 11,872.2] mmHg/mL, *p* < 0.001; RV-PVA 2,176.1 [2,046.3, 2,885.1] to 1,960.2 [1,835.6, 2,269.0] mmHg/mL, *p* = 0.002), with stable SW/PVA ratios. The acute haemodynamic effects of TAVI for severe symptomatic AS extend beyond the LV. Patients experienced bi-ventricular unloading, improved ventriculo-arterial coupling and reduced myocardial metabolic demand.

**Graphical abstract:**

**Figure:**
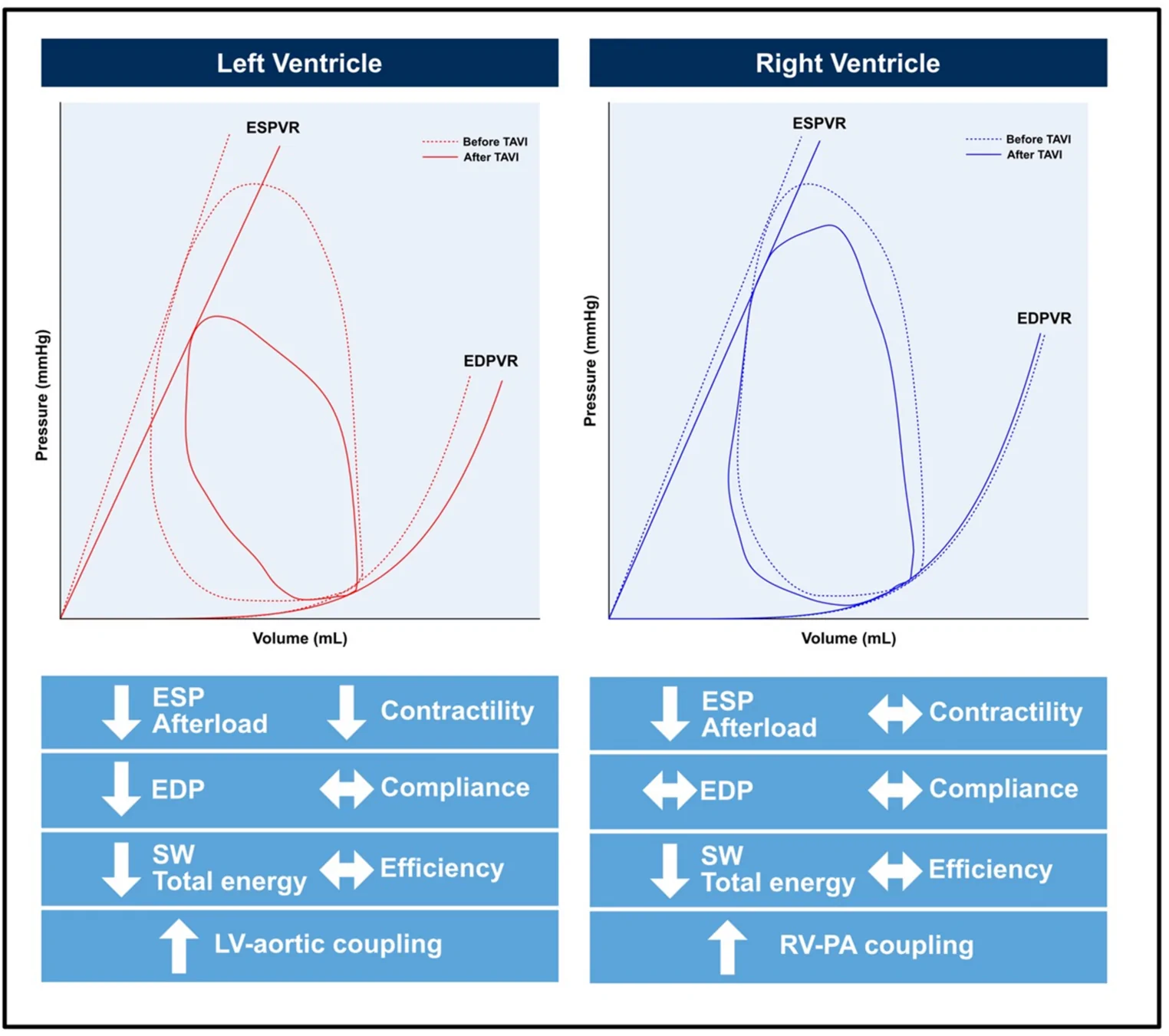
Schematic illustration of changes in bi-ventricular pressure-volume loops following TAVI, representing cohort-averaged data rather than direct measurements, together with the key study findings. EDP: end-diastolic pressure; EDPVR: end-diastolic pressure-volume relationship; ESP: end-systolic pressure; ESPVR: end-systolic pressure-volume relationship; LV: left ventricle; RV-PA: right ventricle-pulmonary arterial; SW: stroke work; TAVI: transcatheter aortic valve implantation.

**Supplementary Information:**

The online version contains supplementary material available at https://doi.org/10.1007/s12928-026-01295-x.

## Introduction

Severe aortic stenosis (AS) is characterised by a narrowed aortic valve, which impedes the flow of blood from the left ventricle (LV) into the systemic circulation. The ensuing chronic pressure overload can be transmitted through the vasculature and via inter-ventricular interactions, resulting in a spectrum of extra-valvular cardio-pulmonary remodelling [1]. While these compensatory mechanisms initially maintain adequate cardiac output, they occur at the expense of increased energy expenditure and ultimately promote the development of heart failure [2]. Indeed, the presence of maladaptive cardio-pulmonary abnormalities is associated with an increased risk of adverse outcomes following AS interventions [3]. 

Transcatheter aortic valve implantation (TAVI) has a primary goal of altering the disease course by increasing the aortic valve orifice area and reducing transaortic gradients, thereby relieving LV pressure overload, prolonging life and improving health status. Chronic recovery of ventricular function after TAVI plays a crucial role in mediating its favourable impact on long-term clinical outcomes. Consequently, reverse remodelling following TAVI has been extensively investigated, predominantly using non-invasive imaging to assess changes in myocardial chamber dimensions and volumes. Reverse LV remodelling following TAVI is characterised by reductions in LV end-diastolic volume (EDV), LV end-systolic volume (ESV) and LV mass, as well as an increase in LV ejection fraction (EF) [4]. Furthermore, TAVI results in significant reversal of right ventricular (RV) dysfunction, mediated at least in part by reduction in pulmonary pressures [5]. However, despite these insights, invasive studies of acute bi-ventricular changes in hemodynamic physiology before and after TAVI are lacking.

Invasive pressure-volume loop (PVL) analysis is the gold standard for evaluating the mechanical properties of the heart and has been widely used to understand ventricular physiology during various cardiovascular interventions [6]. Prior studies on the early haemodynamic effect of TAVI have focussed primarily on the LV, whilst the acute impact on the RV remains uncharacterised [7]. This represents an important knowledge gap since the bi-ventricular myocardial response to changing loading conditions after TAVI may provide important explanations for the varying prognostic trajectories following the procedure. Therefore, the aim of this mechanistic study was to determine the acute unloading effects of TAVI on bi-ventricular physiology through invasive PVL analysis.

## Methodology

### Study group

Twelve adult patients (> 18 years) with severe symptomatic AS undergoing elective TAVI under conscious sedation were prospectively enrolled at a single centre between June 2023 and April 2025. Severe AS was defined in accordance with current guidelines as a peak aortic jet velocity ≥ 4 m/s, mean aortic valve gradient (MG) ≥ 40 mmHg and aortic valve area (AVA) < 1 cm^2^ (or indexed AVA ≤ 0.6cm^2^/m^2^) [8]. Exclusion criteria were pronounced bundle branch block or rhythm disorders during invasive assessment (i.e., frequent premature ventricular contractions), at least moderate valvular heart disease other than AS, previous valve surgery, implanted permanent pacemaker, history of lung disease, left ventricular ejection fraction (LVEF) < 50%, significant coronary artery disease (CAD), prior myocardial infarction, major post-procedural complications (as defined by Valve Academic Research Consortium-3 [VARC-3] criteria), significant cognitive impairment, or poor transthoracic echocardiography (TTE) image quality [9]. Pre-specified demographic, clinical, laboratory, echocardiographic and procedural data were prospectively collected for all patients. Participants underwent invasive right heart catheterisation (RHC) before TAVI, and PVL assessment both before and after TAVI during the same procedure. The study protocol complied with the principles of the Declaration of Helsinki and was approved by the national ethical review board (Reference: 23/LO/0331). All patients provided written informed consent.

### TAVI procedure

Following Heart Team assessment to determine eligibility, study participants underwent percutaneous transfemoral TAVI with commercially available balloon-expandable or self-expanding bio-prostheses. Selection of prosthesis type and size was guided by cardiac computed tomography angiography in all patients. TAVI device implantation was performed during pacing over a pre-shaped Safari guidewire (Boston Scientific, USA) at the LV apex. Balloon-expandable valves were implanted under rapid ventricular pacing (180–220 bpm), whereas self-expanding valve implantation required lower controlled pacing rates (120–140 bpm). Conscious sedation was achieved using low doses of midazolam (2–5 mg, intravenously) and fentanyl (2 mg/kg, intravenously). Care was taken to ensure a constant level of sedation during pre- and post-procedural measurements.

### Invasive right heart catheterisation and pressure volume loop measurements

**Fig. 1:**
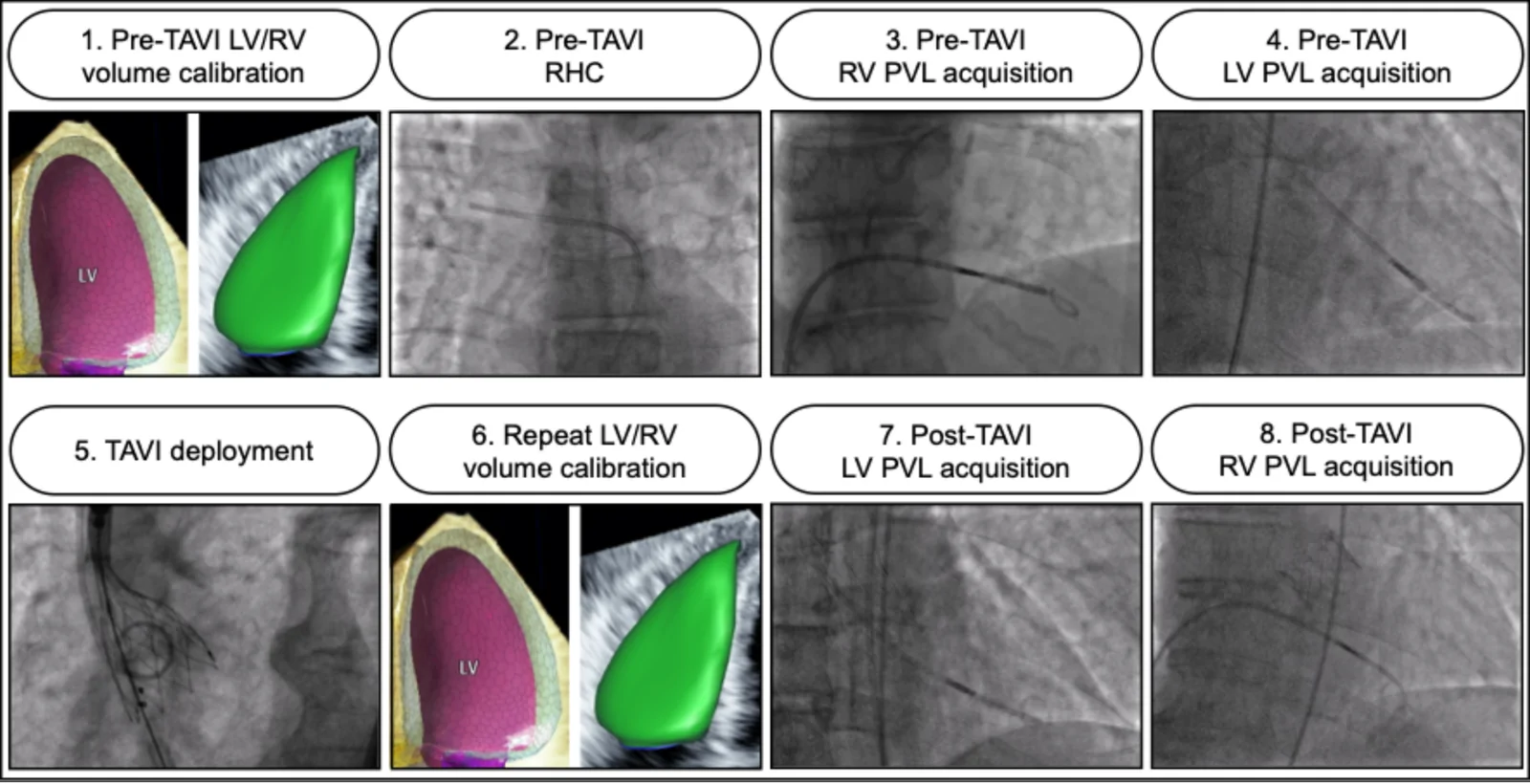
Schematic of the study protocol. Catheter laboratory protocol for bi-ventricular pressure-volume loop acquisition using a conductance catheter before and after transcatheter aortic valve implantation, including baseline right heart catheterisation and echocardiographic volume calibration. LV: left ventricle; PVL: pressure-volume loop; RHC: right heart catheterisation; RV: right ventricle; TAVI: transcatheter aortic valve implantation

Prior to TAVI, all patients underwent invasive RHC via femoral venous access using a 7 F fluid filled Swan-Ganz catheter (Edward Lifesciences, USA) to characterise the presence and mechanism of pulmonary hypertension (PH) (Supplementary Methods). Real-time PVL acquisitions were performed using a 7 F pigtail conductance catheter (CD Leycom, Zoetermeer, Netherlands), according to the study protocol shown in Fig. 1, as previously described [10]. Pressure calibration was performed with the catheter tip immersed in normal saline at heart level, and volume calibration with bi-ventricular volumes obtained using three-dimensional (3D) TTE acquired immediately before and 15 min after TAVI deployment (Supplementary Methods). After calibration, the PVL catheter was advanced into the LV and then the RV under fluoroscopic guidance via femoral arterial and venous access, respectively. After connection to a dedicated signal processor (Inca, CD Leycom, Zoetermeer, Netherlands), segmental volume signals and PVLs were visually assessed for angular shape, counter-clockwise orientation and absence of twists to enable further analysis. The PVL catheter was repositioned if < 4 segments were suitable for analysis, or if signal quality was unstable. After confirmation of a stable catheter position at the ventricular apex without induction of premature ventricular contractions, ventricular volume, pressure and the electrocardiogram (ECG) were recorded simultaneously (2 × 15s) under steady-state conditions at end-expiration. Beat-by-beat variables were averaged across all cardiac cycles after exclusion of aberrant beats and artefacts, and a dataset considered representative if it contained high-quality PVLs of at least 15 heart beats.

### Pressure volume loop analysis

**Fig. 2:**
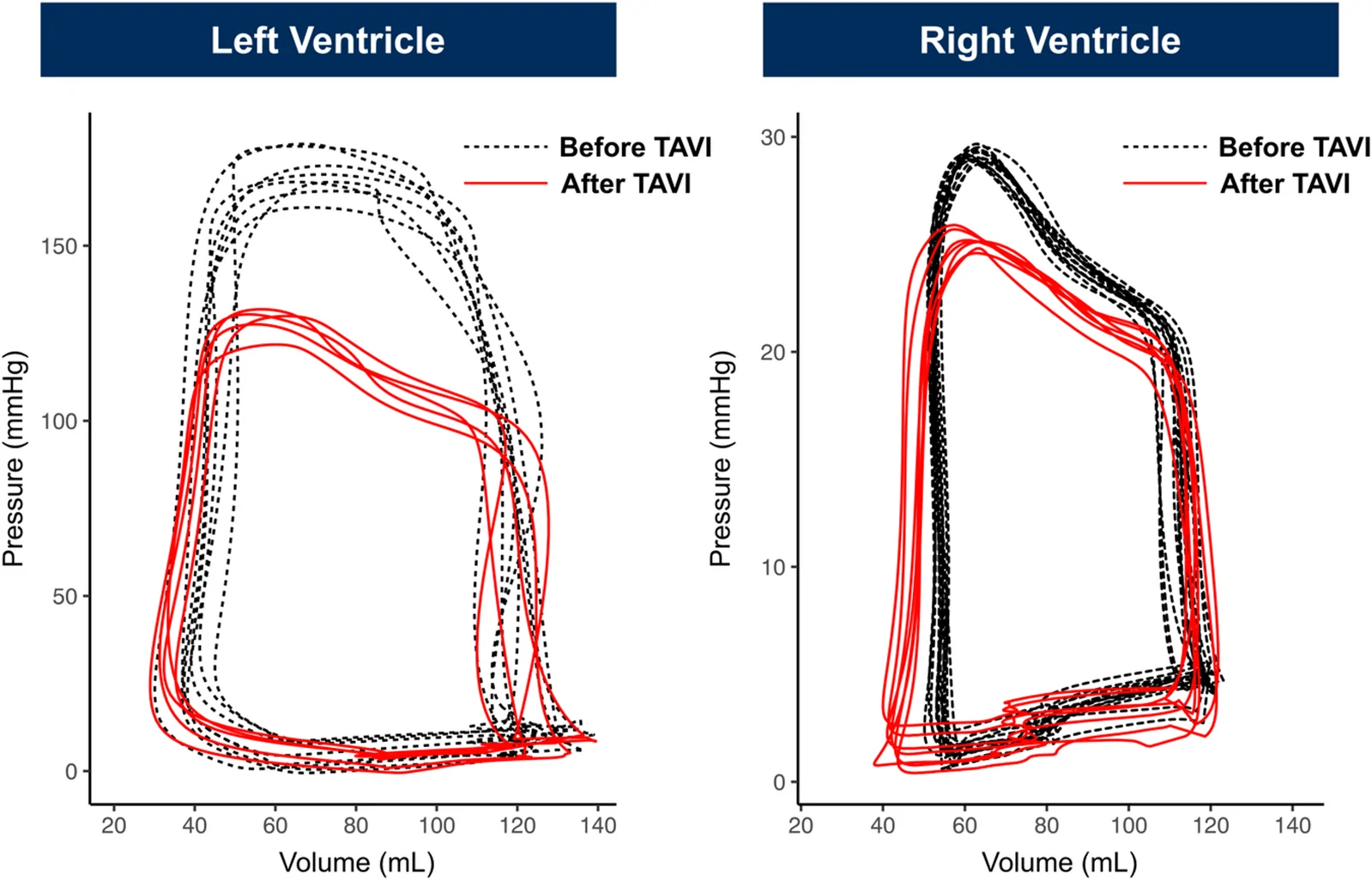
Study participant pressure-volume loops. Representative bi-ventricular pressure-volume loops acquired from a participant before and after transcatheter aortic valve implantation. TAVI: transcatheter aortic valve implantation

PVLs were analysed using dedicated ConductNT software (CD Leycom, Zoetermeer, Netherlands) by a single study investigator (V.A.) blinded to patient clinical characteristics [6]. A representative example of pre- and post-TAVI bi-ventricular PVLs obtained from a study participant is shown in Fig. 2 (additional examples are provided in Supplementary Fig. 1 [Supplementary Material]). Single erroneous loops were excluded after beat-by-beat visual inspection, and correct identification of end-systole and end-diastole was verified manually against the ECG. Volume calibration was performed prior to analysis and all reported volumes were indexed to body surface area (BSA). PVL analysis included indices of systolic and diastolic function, ventricular energetics and ventriculo-arterial coupling.

Reflecting global cardiac haemodynamics, the following parameters were analysed: heart rate (HR, beats/min), EDV index (EDVi), ESV index (ESVi), EF ([EDV – ESV]/EDV), stroke volume (SV; EDV-ESV), stroke volume index (SVi; SV/BSA), cardiac index (CI; SV × HR/BSA), invasively-recorded ventricular end-systolic pressure (ESP) and ventricular end-diastolic pressure (EDP).

To characterise systolic function, the end-systolic pressure-volume relationship (ESPVR) was derived by single-beat estimation as the ratio of ventricular ESP to ESV, with the volume-axis intercept fixed at zero (V_0_), as previously described [11]. Ventricular pressure and volume were recorded continuously, and ESP was defined as the point on the pressure-volume loop at which the instantaneous pressure-volume ratio reached its maximum, with ESPVR then defined as the line connecting the origin (V_0_) to this point. Subsequently, end-systolic elastance (E_es_) was calculated as the gradient of the ESPVR, representing a load-independent index of contractility [12]. Preload-recruitable stroke work (PRSW), determined from stroke work (SW) divided by EDV, was used to characterise the myocardial contractile response to increasing preload, thus serving as a marker of contractile performance. The maximal rate of increase in systolic pressure over time (dP/dt+) was described as a marker of load-dependent contractility.

To assess diastolic properties, the end-diastolic pressure-volume relationship (EDPVR) was derived using a single-beat approach [13]. Relative comparisons of chamber compliance were derived using the EDV at a pre-defined EDP of 30 mmHg (V30) for the LV, and 10 mmHg (V10) for the RV. The maximum rate of decrease in diastolic pressure over time (dP/dt-) was calculated as a pre-load dependent marker of relaxation. The time constant of isovolumetric relaxation Tau (τ) was defined as the time from dP/dt- until EDP reaches half the value of dP/dt-.

Myocardial energetics were quantified using SW and pressure volume area (PVA) [14]. SW refers to the external mechanical work performed by the ventricle to eject blood into the arterial circulation, and was quantified by the area enclosed by the PVL. PVA represents the total mechanical energy generated by the ventricle per beat and was calculated as the sum of SW and residual myocardial potential energy (PE) stored at the end of contraction (Supplementary Methods). Subsequently, haemodynamic efficiency is expressed as SW/PVA.

The effective arterial elastance (E_a_), indicating total ventricular afterload, was quantified as the ratio of ESP to SV [15]. Ventriculo-arterial coupling, representing the interaction between contractility and afterload, was conventionally expressed as E_a_/E_es_ in the LV and E_es_/E_a_ in the RV [16]. 

### Statistical analysis

Variables are presented as median (interquartile range [IQR]) or number (%). Given the small sample size, the non-parametric Wilcoxon signed-rank test was used to compare changes in PVL parameters before and after TAVI. All analyses were performed using R version 4.4.0 (R Foundation for Statistical Computing, Vienna, Austria) and statistical significance was defined as *p* < 0.05.

## Results

### Patient characteristics

The clinical characteristics of the study population are presented in Table 1. A total of 12 patients (median age 84.1 years [77.1, 87.2]; *n* = 3, 25.0% female) underwent conductance catheter measurements. Prior to TAVI, all participants had severe isolated AS (AVA 0.6 cm^2^ [0.5, 0.7]; transaortic MG 42.5 mmHg [41.0, 45.0]) and generally preserved bi-ventricular function (LVEF: 60.3% [57.6, 62.8]; TAPSE: 18.3 mm [18.1, 20.3]). All patients were symptomatic with median New York Heart Association (NYHA) class 2 (2, 3) and Kansas City Cardiomyopathy Questionnaire overall summary (KCCQ-OS) score of 47.9 (39.8, 56.0). Baseline RHC showed a mean PAP of 15.5 mmHg (13.7, 19.3), with 3 patients (25.0%) meeting the criteria for isolated post-capillary PH. TAVI was performed using balloon-expandable prostheses in 9 patients (75.0%). Post-procedure, the AVA was 1.9 cm^2^ (1.9, 2.2) and the transvalvular MG was 8.5 mmHg (5.0, 9.2). There were no peri-procedural complications or ≥mild para- or trans-valvular regurgitation. Pre- and post-TAVI LV PVL analysis was feasible in all patients, although stable haemodynamic signals for RV PVL could not be obtained in 2 patients (16.7%), who were excluded from further RV analysis. Acute post-TAVI changes in bi-ventricular haemodynamic parameters are presented in Table 2.

**Table 1:** Patient characteristics

Variable	Study cohort (*n* = 12)
**Clinical**	
Age (years)	84.1 (77.1, 87.2)
Female sex (%)	3 (25.0%)
Atrial fibrillation (%)	4 (33.3%)
Hypertension (%)	8 (66.7%)
Hypercholesterolaemia (%)	4 (33.3%)
Diabetes mellitus (%)	3 (25.0%)
Current/ex-smoker (%)	4 (33.3%)
BMI (kg/m^2^)	26.0 (23.0, 28.3)
**Laboratory**	
Hb (g/L)	134.0 (121.8, 139.3)
eGFR (mL/min/1.73 m²)	66.5 (61.8, 70.5)
NT-proBNP (pg/mL)	817.5 (543.5, 1,360.0)
**Transthoracic echocardiography**	
AVA (cm^2^)	0.6 (0.5, 0.7)
AV max (m/s)	4.2 (4.1, 4.5)
AV MG (mmHg)	42.5 (41.0, 45.0)
Simpson’s LVEF (%)	60.3 (57.6, 62.8)
LV mass (g)	204.1 (164.5, 229.8)
E/e’	14.9 (12.8, 17.7)
LA ESV (mL)	91.1 (67.8, 95.8)
PASP (mmHg)	31.2 (28.2, 39.2)
TAPSE (mm)	18.3 (18.1, 20.3)
RA ESV (mL)	39.7 (33.1, 77.8)
**Symptoms**,** health status and function**	
NYHA Class	2.0 (2.0, 3.0)
KCCQ-OS	47.9 (39.8, 56.0)
KCCQ-CS	52.1 (43.2, 60.7)
**Right heart catheterisation**	
RAP (mmHg)	3.5 (2.0, 5.3)
RVSP (mmHg)	27.0 (24.8, 34.0)
RVDP (mmHg)	3.5 (3.0, 6.3)
mPAP (mmHg)	15.5 (13.7, 19.3)
PCWP (mmHg)	8.5 (7.0, 12.3)
TPG (mmHg)	6.5 (5.5, 8.0)
PVR (WU)	1.2 (1.1, 1.8)
**Procedure**	
Balloon-expandable prosthesis (%)	9 (75.0%)
Device size (mm)	26 (23, 26)
Post-TAVI AVA (cm^2^)	1.9 (1.9, 2.2)
Post-TAVI AV max (m/s)	2.0 (1.7, 2.2)
Post TAVI AV MG (mmHg)	8.5 (5.0, 9.2)
Post TAVI ≥mild AR (%)	0 (0%)

AR: aortic regurgitation; AV: aortic valve; AVA: aortic valve area; BMI: body mass index; eGFR: estimated glomerular filtration rate; ESV: end-systolic volume; Hb: haemoglobin; KCCQ-OS: Kansas City Cardiomyopathy Questionnaire overall summary score; KCCQ-CS: Kansas City Cardiomyopathy Questionnaire clinical summary score; LA: left atrium; LV: left ventricle; LVEF: left ventricular ejection fraction; MG: mean gradient; mPAP: mean pulmonary artery pressure; NT-proBNP: N-terminal pro-B-type natriuretic peptide; NYHA: New York Heart Association; PASP: pulmonary artery systolic pressure; PCPW: pulmonary capillary wedge pressure; PVR: pulmonary vascular resistance; RA: right atrium; RAP: right atrial pressure; RVDP: right ventricular diastolic pressure; RVSP: right ventricular systolic pressure; TAPSE: tricuspid annular plane systolic excursion; TAVI: transcatheter aortic valve implantation; TPG: trans-pulmonary gradient

**Table 2:** Pressure-volume loop-derived haemodynamic indices

Variable	Left ventricle (*n* = 12)			Right ventricle (*n* = 10)		
	Before TAVI	After TAVI	*P* valve	Before TAVI	After TAVI	*p* valve
**Basic haemodynamics**						
Heart rate (bpm)	67.1 (64.3, 81.0)	71.0 (62.1, 79.9)	0.622	67.7 (64.6, 85.7)	77.3 (64.0, 87.9)	0.432
EDVi (mL/m^2^)	64.5 (59.7, 70.7)	64.2 (58.4, 69.0)	**0.004**	60.7 (54.2, 68.8)	59.1 (52.8, 67.4)	**0.004**
ESVi (mL/m^2^)	25.0 (23.3, 27.4)	22.5 (21.3, 24.8)	**< 0.001**	27.3 (25.5, 36.5)	25.0 (22.7, 31.8)	**0.002**
SVi (mL/m^2^)	39.8 (37.9, 43.5)	41.3 (39.2, 44.1)	**< 0.001**	31.2 (28.4, 34.0)	32.9 (29.9, 35.3)	**0.009**
3D EF (%)	61.5 (58.5, 62.5)	63.5 (62.3, 65.5)	**< 0.001**	52.1 (47.0, 53.8)	55.5 (52.7, 57.3)	**0.002**
CI (L/min/m^2^)	2.7 (2.4, 3.1)	2.6 (2.3, 3.5)	0.569	2.2 (2.1, 2.4)	2.6 (1.9, 2.7)	0.160
**Systolic function**						
ESP (mmHg)	151.8 (133.3, 175.1)	114.6 (97.0, 127.3)	**< 0.001**	24.9 (23.5, 39.0)	22.9 (21.7, 27.1)	**0.002**
E_es_ (mmHg/mL)	3.2 (2.5, 3.6)	2.6 (2.2, 3.1)	**0.021**	0.5 (0.5, 0.5)	0.5 (0.4, 0.5)	0.759
dP/dt+ (mmHg/s)	1,513.7 (1,317.0, 1,834.4)	1,225.9 (956.3, 1,378.2)	**< 0.001**	320.0 (300.3, 406.1)	304.5 (262.8, 350.2)	0.105
PRSW (mmHg)	98.4 (76.6, 130.1)	68.5 (56.4, 77.8)	**< 0.001**	14.9 (11.3, 15.6)	12.5 (11.1, 13.4)	0.064
**Diastolic function**						
EDP (mmHg)	15.5 (10.2, 18.5)	8.5 (7.3, 10.6)	**0.009**	3.6 (2.6, 4.4)	3.0 (1.9, 4.4)	0.846
Tau (ms)	30.2 (28.0, 34.9)	35.5 (29.0, 39.7)	0.433	29.3 (26.1, 37.3)	28.9 (24.9, 32.3)	0.813
dP/dt- (mmHg/s)	-1,553.8 (-1,760.9, -1,135.0)	-1,096.6 (-1,300.3, -809.0)	**0.007**	-261.4 (-310.5, -237.5)	-247.1 (-264.0, -216.6)	0.084
V30 (LV) or V10 (RV) (mL)	140.0 (132.5, 152.5)	144.3 (135.3, 155.5)	0.110	143.2 (121.6, 155.7)	138.4 (126.9, 150.3)	0.625
**Energetics**						
SW (mmHg.mL)	11,915.0 (9,727.5, 15,288.7)	7,360.5 (6,937.0, 9,113.1)	**< 0.001**	1,506.9 (1,420.5, 1,676.2)	1,418.1 (1,250.8, 1,510.3)	**0.020**
PVA (mmHg.mL)	16,188.5 (13,171.5, 18,726.5)	9,674.2 (9,185.9, 11,872.2)	**< 0.001**	2,176.1 (2,046.3, 2,885.1)	1,960.2 (1,835.6, 2,269.0)	**0.002**
Haemodynamic efficiency (%)	76.2 (73.2, 80.1)	76.3 (74.7, 77.4)	0.791	68.7 (60.7, 74.1)	72.0 (62.3, 75.7)	0.160
**Ventriculo-arterial coupling**						
E_a_ (mmHg/mL)	2.1 (1.8, 2.3)	1.4 (1.3, 1.7)	**< 0.001**	0.5 (0.4, 0.6)	0.4 (0.3, 0.4)	**0.006**
E_a_/E_es_ (LV) or E_es_/E_a_ (RV)	0.7 (0.6, 0.7)	0.6 (0.5, 0.6)	**0.003**	1.1 (0.9, 1.3)	1.3 (1.1, 1.4)	**0.006**

CI: cardiac index; dP/dt+: maximum rate of pressure change; dP/dt-: minimum rate of pressure change; E_a_: effective arterial elastance; EDP: end-diastolic pressure; EDVi: end-diastolic volume index; EF: ejection fraction; E_es_: end-systolic elastance; ESP: end-systolic pressure; ESVi: end-systolic volume index; LV: left ventricle; PRSW: preload-recruitable stroke work; PVA: pressure-volume area; RV: right ventricle; SVi: stroke volume index; SW: stroke work; TAVI: transcatheter aortic valve implantation; V10: ventricular volume at end-diastolic pressure of 10mmHg; V30: ventricular volume at end-diastolic pressure of 30mmHg; 3D: 3-dimensional

### Effect of TAVI on volumes and ejection fraction

LV unloading with TAVI was associated with significant reductions in ESVi (LV: *p* < 0.001; RV: *p* = 0.002) and EDVi (LV: *p* = 0.004; RV: *p* = 0.004), alongside significant increases in SVi (LV: *p* < 0.001, RV: *p* = 0.009), driven by the relatively larger fall in ESVi. Consequently, significant increases in EF were seen in both the LV (*p* < 0.001) and RV (*p* = 0.002). No significant changes in HR or CI were observed in either ventricle (both *p* > 0.05).

### Effect of TAVI on systolic function

**Fig. 3:**
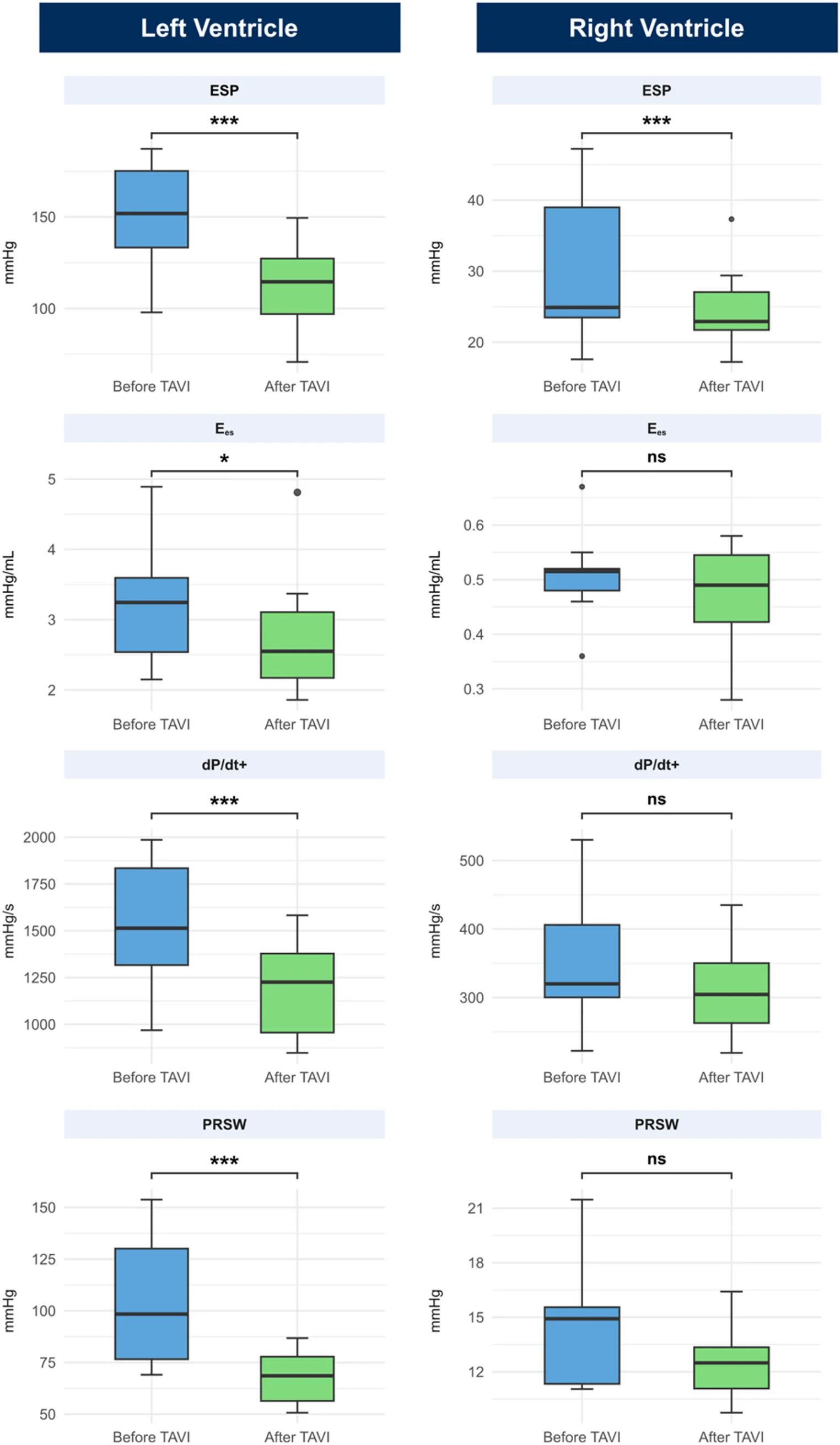
Intra-procedural changes in systolic function. Box-and-whisker plots: box length represents the IQR; horizontal box line represents the median; whiskers represent the maximum and minimum values excluding outliers (shown as dots); * denotes *p* < 0.05, **p < 0.01, ***p < 0.001, ns: non-significant (*p* > 0.05). E_es_: end-systolic elastance; ESP: end-systolic pressure; IQR: inter-quartile range; PRSW: preload-recruitable stroke work; TAVI: transcatheter aortic valve implantation

Changes in bi-ventricular systolic function following TAVI are presented in Fig. 3. In the LV, there was a significant fall in ESP (151.8 mmHg [133.3, 175.1] to 114.6 mmHg [97.0, 127.3], *p* < 0.001), accompanied by significant reduction in indices of myocardial contractility, including E_es_ (3.2 mmHg/mL [2.5, 3.6] to 2.6 mmHg/mL [2.2, 3.1], *p* = 0.021), dP/dt+ (*p* < 0.001) and PRSW (*p* < 0.001). In the RV, ESP also fell significantly (24.9 mmHg [23.5–39.0] to 22.9 mmHg [21.7–27.1], *p* < 0.001), but no significant changes were observed in E_es_ (*p* = 0.759), dP/dt+ (*p* = 0.105) or PRSW (*p* = 0.064).

### Effect of TAVI on diastolic function

**Fig. 4:**
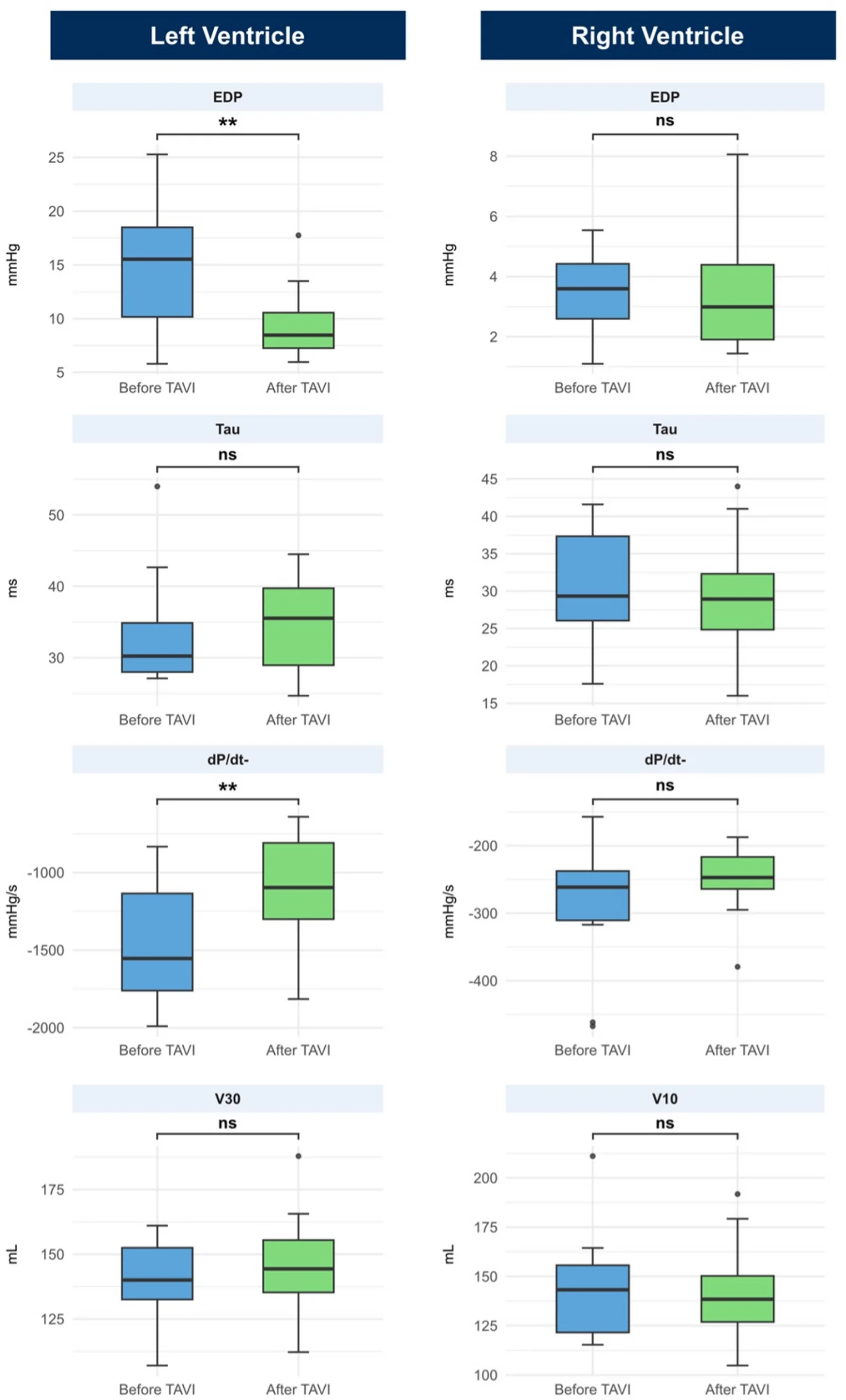
Intra-procedural changes in diastolic function. Box-and-whisker plots: box length represents the IQR; horizontal box line represents the median; whiskers represent the maximum and minimum values excluding outliers (shown as dots); * denotes *p* < 0.05, **p < 0.01, ***p < 0.001, ns: non-significant (*p* > 0.05). EDP: end-diastolic pressure; IQR: inter-quartile range; TAVI: transcatheter aortic valve implantation; V10: ventricular volume at end-diastolic pressure of 10mmHg; V30: ventricular volume at end-diastolic pressure of 30mmHg

The acute impact of TAVI on LV and RV diastolic function is presented in Fig. 4. In the LV, a significant fall in EDP (15.5 mmHg [10.2, 18.5] to 8.5 mmHg [7.3, 10.6], *p* = 0.009) was observed, while compliance did not change significantly (V30: *p* = 0.110). Reflecting impairment of diastolic function, the absolute value of the minimum rate of pressure change (dP/dt-) fell significantly (*p* = 0.007), albeit with no change in the isovolumic relaxation parameter τ (*p* = 0.433). In the RV, no significant changes were observed in EDP (*p* = 0.846), V10 (*p* = 0.625), dP/dt- (*p* = 0.084) or τ (*p* = 0.813).

### Effect of TAVI on myocardial energetics

**Fig. 5:**
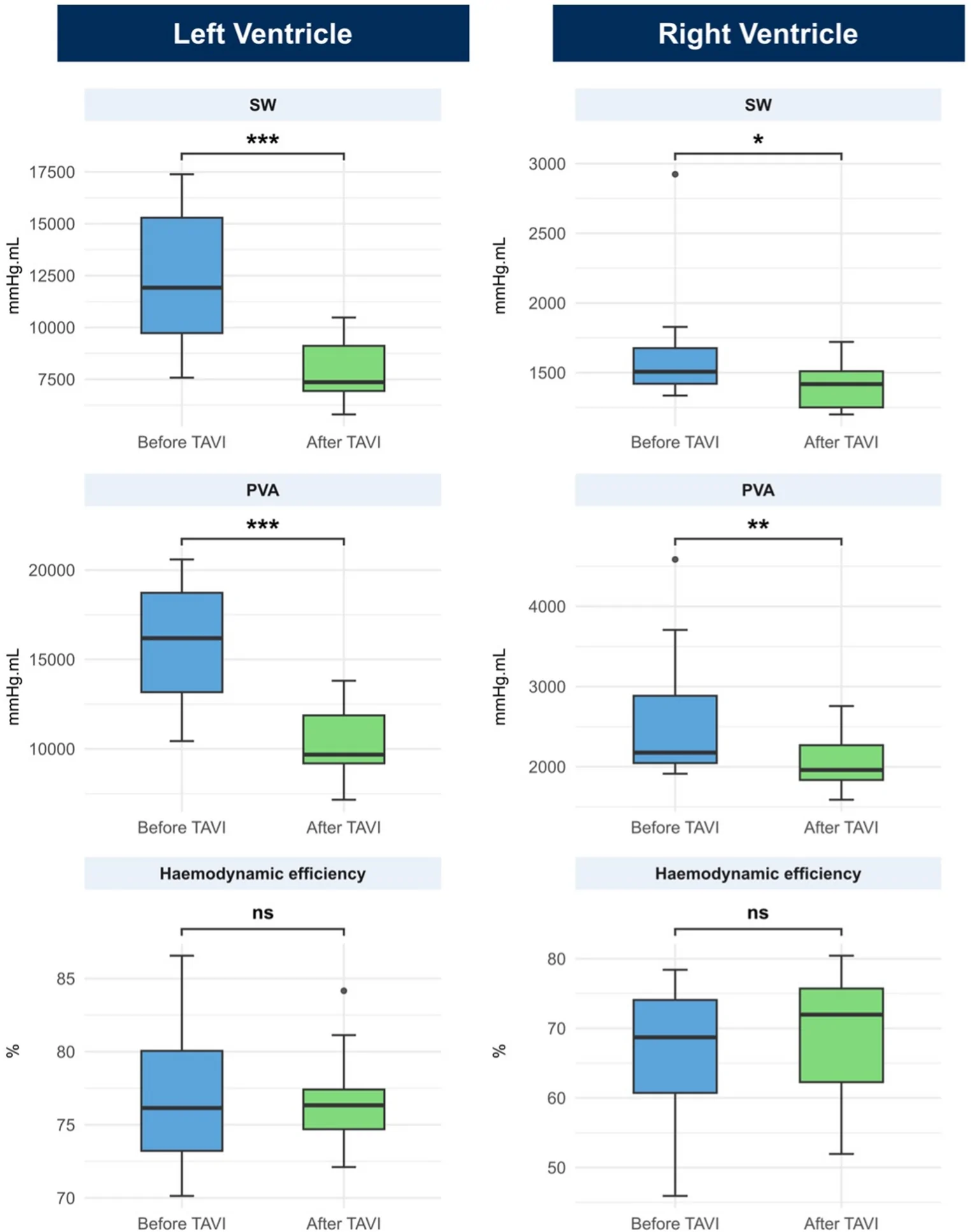
Intra-procedural changes in myocardial energetics. Box-and-whisker plots: box length represents the IQR; horizontal box line represents the median; whiskers represent the maximum and minimum values excluding outliers (shown as dots); * denotes *p* < 0.05, **p < 0.01, ***p < 0.001, ns: non-significant (*p* > 0.05). IQR: inter-quartile range; PVA: pressure-volume area; SW: stroke work; TAVI: transcatheter aortic valve implantation

Changes in ventricular energetics following TAVI are presented in Fig. 5. In the LV, there were significant reductions in SW (11,915.0 mmHg.mL [9,727.5, 15,288.7] to 7,360.5 mmHg.mL [6,937.0, 9,113.1], *p* < 0.001) and PVA (16,188.5 mmHg.mL [13,171.5, 18,726.5] to 9,674.2 mmHg.mL [9,185.9, 11,872.2], *p* < 0.001), with no significant change in haemodynamic efficiency (*p* = 0.791). Similarly, in the RV, there were significant reductions in both SW (1,506.9 mmHg.mL [1,420.5, 1,676.2] to 1,418.1 mmHg.mL [1,250.8, 1,510.3], *p* = 0.020) and PVA (2,176.1 mmHg.mL [2,046.3, 2,885.1] to 1,960.2 mmHg.mL [1,835.6, 2,269.0], *p* = 0.002), with no significant change in haemodynamic efficiency (*p* = 0.160).

### Effect of TAVI on ventriculo-arterial coupling

**Fig. 6:**
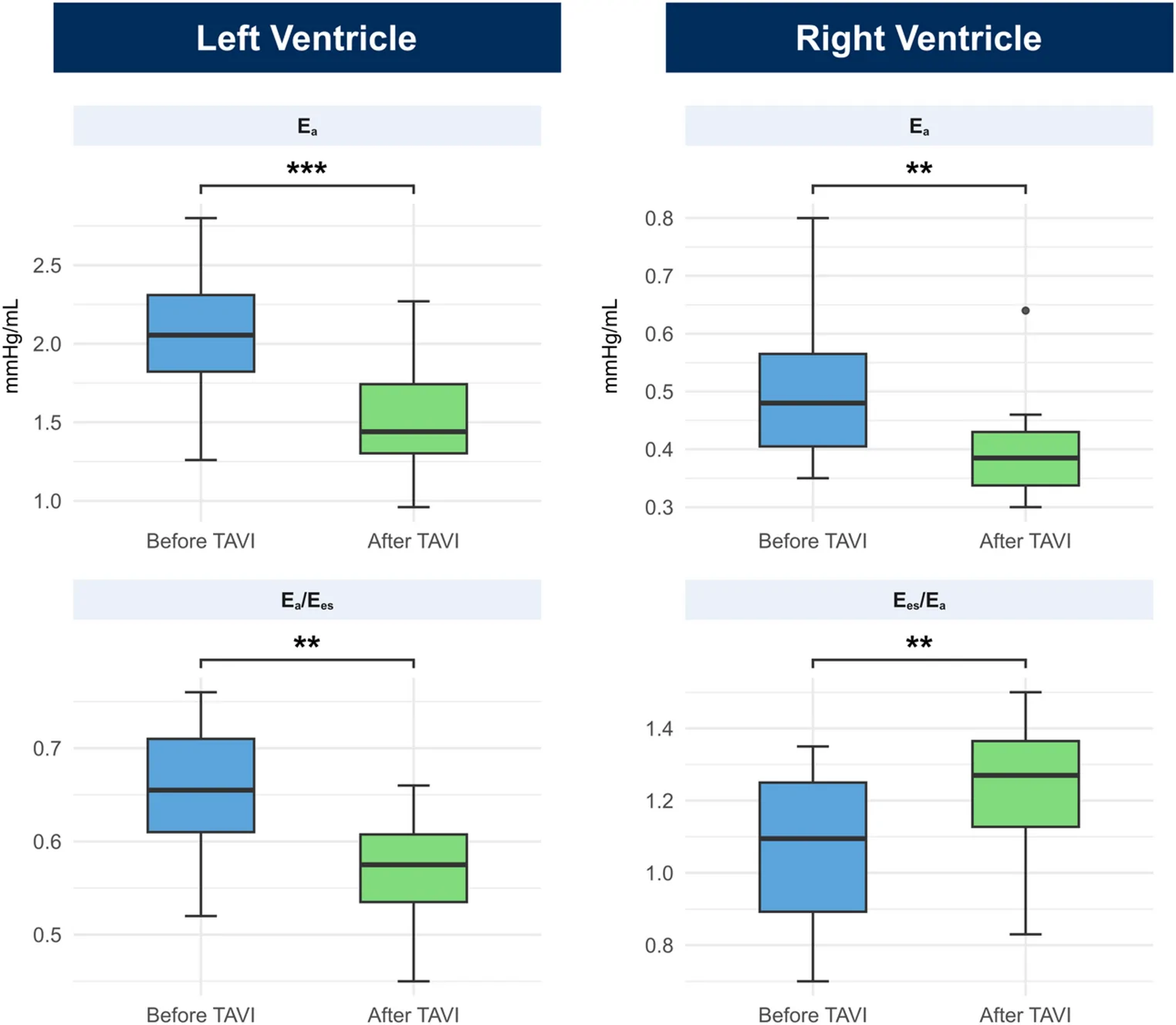
Intra-procedural changes in ventriculo-arterial coupling. Box-and-whisker plots: box length represents the IQR; horizontal box line represents the median; whiskers represent the maximum and minimum values excluding outliers (shown as dots); * denotes *p* < 0.05, **p < 0.01, ***p < 0.001, ns: non-significant (*p* > 0.05). E_a_: effective arterial elastance; E_es_: end-systolic elastance; IQR: inter-quartile range; TAVI: transcatheter aortic valve implantation

Changes in ventriculo-arterial coupling after TAVI are presented in Fig. 6. In the LV, there was a significant fall in E_a_ (2.1 mmHg/mL [1.8, 2.3] to 1.4 mmHg/mL [1.3, 1.7], *p* < 0.001) that outweighed the corresponding fall in E_es_, resulting in an overall improvement in LV ventriculo-arterial coupling (E_a_/E_es_: 0.7 [0.6, 0.7] to 0.6 [0.5, 0.6], *p* = 0.003). A similar pattern was seen in the RV, with a significant fall in E_a_ (0.5 mmHg/mL [0.4, 0.6] to 0.4 mmHg/mL [0.3, 0.4], *p* = 0.006) driving a significant improvement in RV ventriculo-arterial coupling (E_es_/E_a_: 1.1 [0.9, 1.3] to 1.3 [1.1, 1.4], *p* = 0.006).

## Discussion

This is the first study to invasively characterise acute bi-ventricular changes in PVL-derived haemodynamic indices after TAVI for severe symptomatic AS. Our findings demonstrate that TAVI is associated with: (1) an immediate acute decrease in LV ESP and EDP; (2) an early decline in load-dependent (dP/dt+) and load-independent (E_es_, PRSW) measures of LV contractility; (3) bi-ventricular improvement in ventriculo-arterial coupling, driven by a large fall in afterload (E_a_); (4) no change in LV and RV passive elastic properties (as assessed by compliance), although the LV showed a reduction in EDP and prolongation of active relaxation (dP/dt-); (5) a fall in bi-ventricular SW and PVA, with no immediate change in total haemodynamic efficiency (Graphical Abstract).

Taken together, these salient haemodynamic changes demonstrate the sequential effects of TAVI for severe AS on bi-ventricular function. Previous literature concerning peri-procedural haemodynamic assessment using invasive PVL analysis in TAVI recipients has been predominantly confined to the LV. By incorporating additional RV evaluation in the present study, we provide a novel overview of acute changes in whole-heart physiology following TAVI. Broadly, successful relief of LV afterload reduced LV ESP and EDP, thereby lowering LV filling pressure, with sequential transmission to the pulmonary vascular bed and reduction of RV afterload. Despite an acute fall in LV contractility, the overall reduction in bi-ventricular afterload resulted in improved ventriculo-arterial coupling, suggesting an early improvement in global cardiovascular performance. The downward shift of both LV and RV PVLs was accompanied by reduced SW and PVA, indicating lower myocardial metabolic demand and oxygen consumption. The long-term clinical implications of the acute bi-ventricular haemodynamic alterations observed after TAVI remain poorly understood. Because invasive PVL analysis is technically demanding, requires specialist expertise, and is costly and time-consuming, it remains largely restricted to research settings, and large-scale clinical studies have not yet been conducted. However, given that TAVI is inherently invasive, peri-procedural PVL analysis could serve as a valuable adjunct to characterise the acute haemodynamic response, without undue additional risk to patients, which may help identify novel invasive biomarkers for improved prediction of mortality and functional outcomes. Nevertheless, important barriers to widespread implementation remain, including the need for accessible and user-friendly analysis software, which would facilitate broader adoption of invasive PVL analysis in the TAVI field.

Historically, the physiology of the RV has been less extensively investigated than that of the LV. In this regard, our study demonstrates that the unloading effect of TAVI causes acute changes in RV haemodynamics, extending beyond the LV. Notably, there was an immediate reduction in RV afterload, indicating a lower force required for the RV to eject blood into the pulmonary circulation. This was accompanied by improved RV-pulmonary arterial (RV-PA) coupling, reduced RV metabolic demand and an increase in RVEF, although load-independent measures of RV contractility remained unchanged. Interestingly, these changes occurred in the context of preserved baseline RVEF, suggesting that these patients had a degree of ineffective RV work and sub-clinical RV dysfunction prior to intervention. Collectively, these results provide new insights into the acute haemodynamic changes following TAVI and reinforce the importance of examining reverse remodelling in the context of bi-ventricular function, given the close interventricular interaction.

In the context of the RV, one prior case report provided an elegant description of PVL changes across all four cardiac chambers following TAVI for severe bicuspid AS, identifying a reduction in post-procedural RV SW consistent with the findings of the present study [17]. Additionally, our results are consistent with a previous study in 44 patients that used RHC and TTE to demonstrate the acute effect of TAVI in improving PVR as well as RV SW, tricuspid lateral annulus systolic velocity (S′) and RV-PA coupling [18]. However, this study found that pulmonary pressures did not change immediately, suggesting that improvements in RV-PA coupling were predominantly mediated through improved RV function, potentially via LV-RV systolic interaction. This demonstrates that the acute mechanism of RV-PA coupling improvement following TAVI may be influenced by RV- and PA-predominance. Furthermore, the aforementioned study included patients with a high burden of baseline RV dysfunction (50%) and PH (61%), suggesting that the specific mechanism may also depend on the extent of baseline extra-valvular remodelling. Indeed, non-invasive studies show that PH [19] and RV dysfunction [20] can improve in the long-term following TAVI, and that these improvements are associated with more favourable prognosis. However, reliable predictors of persistent right-sided cardio-pulmonary dysfunction after TAVI are scarce and it remains unclear whether improvement in RV-PA coupling through RV- or PH-predominant mechanisms influences long-term outcomes. Larger haemodynamic studies with long-term follow-up, potentially facilitated by the development of non-invasive PVL reconstruction techniques to improve scalability, are needed to clarify the clinical implications of these early haemodynamic changes.

In the context of the existing evidence related to the LV, the finding that LV systolic function declined acutely after TAVI is consistent with the latest and largest study to date by van den Enden et al., which evaluated peri-procedural invasive LV mechanics in 61 TAVI recipients and demonstrated reductions in dP/dt + and E_es_ immediately after intervention [21]. Similarly, Yin et al. reported an acute decrease in LV dP/dt + and PRSW, with no significant change in E_es_, in 19 AS patients treated with TAVI [7]. Reductions in dP/dt+, PRSW and E_es_ following TAVI have also been identified by Seppelt et al. in a smaller study of 8 AS patients [22]. Corroborating our findings of possible acute deterioration of LV diastolic function immediately after TAVI, Yin et al. reported that dP/dt- became less negative and Seppelt et al. observed an additional increase in LV relaxation time constant τ.

There are several potential explanations for these acute changes in LV contractility after TAVI. As patients with severe AS and preserved LVEF recruit their contractile reserve to overcome increased afterload, correction of the central pathophysiological element of AS will reduce this dependence, resulting in an acute relative reduction in indices of intrinsic contractility and relaxation [23]. Moreover, a degree of myocardial injury is common after TAVI, often reflected biochemically by a rise in plasma cardiac biomarkers, which may acutely impair contractile performance [24]. In keeping with this, a greater degree of myocardial injury is associated with reduced improvement in LVEF following TAVI [25]. Furthermore, rapid ventricular pacing during valve implantation has long been known to be responsible for transient myocardial stunning and peri-procedural myocardial injury. However, van den Enden *at al.* demonstrated that the decline in LV contractility following TAVI was independent of device type, use of pre-/post-dilatation or rapid ventricular pacing, and baseline LVEF [21]. The development of bundle branch block is another contributing factor to reduced improvement in LVEF and LV strain following TAVI, although our study excluded patients with new conduction abnormalities. Another potential mechanism could be embolism of debris from the native aortic valve, LV and aorta into the coronary microcirculation during TAVI, thus inducing myocardial ischemia or micro-infarction and acutely affecting contractility [26]. 

Interestingly, the reduction in PVL-derived contractility occurred alongside a small improvement in LVEF. This is unsurprising considering that LVEF is highly load-dependent, and can remain normal despite reduced myocardial contractility, or decline despite preserved contractility when afterload is increased (so called afterload mismatch) [27]. Nevertheless, the early decline in LV contractility following TAVI is likely transient and outweighed by the benefits of afterload reduction and reverse remodelling, as evidenced by longer-term post-procedural improvements in LVEF and diastolic function [28]. However, acknowledging the fundamental limitations of LVEF, immediate changes in LV contractility following TAVI may be more accurately assessed using more load-independent measures, such as myocardial strain. Indeed, the acute change in LV global longitudinal strain after TAVI is superior to LVEF in predicting major adverse cardiac events [29]. 

Despite an acute reduction in LV contractility, we observed improvements in ventriculo-arterial coupling after TAVI, driven primarily by a large fall in LV afterload, indicating early recovery of cardiovascular work transmission. Previous studies have yielded conflicting findings in this domain, with an improvement in LV E_a_/E_es_ observed by Yin et al. and Seppelt et al. [7, 22]., but not van den Enden *at al* [21]. Possible explanations for these discrepancies include: (1) small sample sizes; (2) differences in selection criteria, resulting in patient heterogeneity; (3) nuanced inter-patient differences in TAVI deployment and pacing protocols, leading to variable acute reductions in E_es_ and E_a_, the latter being attributable to both valvular and aortic components; (4) varying degrees of intra-procedural sedation/anaesthesia; and 4) heterogeneous methods used to derive ESPVR (single-beat vs. multi-beat; fixed V_0_ vs. variable V_0_). Nevertheless, despite these inconsistencies, our study concurs with van den Enden et al. and Yin et al. in identifying a significant fall in LV metabolic demand after TAVI, as evidenced by reductions in SW and PVA, indicating reduced external mechanical work and myocardial oxygen consumption. More broadly, routine assessment of invasive bi-ventricular haemodynamics in TAVI may help explain fundamental differences in acute post-procedural myocardial response across patient subgroups at different stages of extra-valvular remodelling and ultimately facilitate the development of improved outcome prediction models. Given the technical complexity and the relatively modest sample sizes of most current studies, establishing an international PVL registry to pool available data could open new avenues for improving understanding cardiovascular pathophysiology in TAVI.

### Limitations

While this study offers new insights into the unloading effect of TAVI on acute bi-ventricular haemodynamics, several important limitations warrant acknowledgement. First, this was a small, exploratory, single-centre study and our findings should therefore be considered hypothesis-generating. Moreover, the generalisability of these findings to the wider and more heterogeneous cohort of TAVI recipients should be interpreted with caution. Future PVL studies in TAVI recipients should consider examining differences in patterns of changing bi-ventricular mechanics among different AS phenotypes, as well as in patients at different stages of extra-valvular remodelling (for example, those with and without LV systolic dysfunction, PH, RV dysfunction, concomitant valvular disease and very severe AS). Second, the impact of the observed haemodynamic changes on clinical outcomes was not ascertained and so the clinical significance of our results remains uncertain. Third, although every effort was made to obtain pre- and post-TAVI measurements under consistent procedural conditions, with no ≥mild paravalvular regurgitation observed in any participant, we cannot exclude the potential influence of subtle intra-procedural variations in conscious sedation level, blood viscosity, catheter position, AS severity, valve type/size or rapid ventricular pacing on PVL measurements. Fourth, single-beat estimation of ESPVR assumed a fixed volume-axis intercept (V_0_) at zero, whereas this may vary over time and be subject-specific in vivo. However, this approach is physiologically appropriate, aligns with previous studies [11], and has been shown to be concordant with experimentally measured V_0_ [30]. Furthermore, LV E_es_ calculated using fixed and variable V_0_ were similar at baseline and followed the same trajectory after TAVI in the most recent series [21]. 

## Conclusions

Invasive PVL analysis characterised the acute bi-ventricular haemodynamic changes in patients with severe symptomatic AS undergoing TAVI. LV unloading improved ventriculo-arterial coupling despite an immediate decline in LV contractility, while also reducing RV afterload and enhancing RV-PA coupling. Bi-ventricular improvement in forward-flow haemodynamics, indicating an early increase in the efficiency of global cardiac energy-transfer, was associated with reduction in total mechanical work and myocardial metabolic demand. Future studies are needed to determine whether these acutely beneficial hemodynamic changes translate into improved long-term myocardial reverse remodelling and clinical outcomes.

## Electronic Supplementary Material

Below is the link to the electronic supplementary material.

Supplementary Material 1

## Data Availability

The data and code used in the present study are available upon reasonable request to the corresponding author.
