# Supplementary material for: Acute bi-ventricular haemodynamic changes after transcatheter aortic valve implantation for severe aortic stenosis assessed using invasive pressure-volume loops
Source: Cardiovasc Interv Ther. 2026 May 23;41(4):1109–23. doi: 10.1007/s12928-026-01295-x (PMC13582086; doi:10.1007/s12928-026-01295-x)
Supplement: Supplementary file 1 [file 12928_2026_1295_MOESM1_ESM.docx]

**Acute bi-ventricular haemodynamic changes after transcatheter aortic valve implantation for severe aortic stenosis assessed using invasive pressure-volume loops.**

**Supplementary Material**

**(Cardiovascular Intervention and Therapeutics)**

**All authors:**

Vitaliy Androshchuk^1*^; Edouard Long^1*^; Omar Chehab^1^; Joshua Wilcox^2^; Benedict McDonaugh^2^; Ronak Rajani^3^; Bernard Prendergast^4^; Tiffany Patterson^2^; Simon Redwood^1^.

**Affiliations**:

^1^ School of Cardiovascular Medicine & Sciences, Faculty of Life Sciences & Medicine, King’s

College London, London, United Kingdom.

^2^ Cardiovascular Directorate, St Thomas' Hospital, London, United Kingdom.

^3^ School of Biomedical Engineering and Imaging Sciences, Faculty of Life Sciences & Medicine, King’s College London, London, United Kingdom.

^4^ Cleveland Clinic London, London, United Kingdom.

*Joint first authors.

**Corresponding Author**:

Dr Vitaliy Androshchuk

King’s College London, The Reyne Institute, London, SE1 7EH.

Email: [V.Androshchuk@nhs.net](mailto:V.Androshchuk@nhs.net)

Tel: +447828095450

**Contents**

**Supplementary Material	Page No.**

Supplementary Methods	3

Supplementary Figure 1	4

References	5

**Supplementary Methods**

**Right Heart Catheterisation**

Prior to TAVI, all patients underwent invasive RHC via femoral venous access using a 7F fluid filled Swan-Ganz catheter (Edward Lifesciences, USA) to characterise the presence and mechanism of pulmonary hypertension (PH).[1] After connection to a pressure transducer, the catheter was calibrated with the zero point at the mid-thoracic line to ensure accurate measurements. The balloon-tipped catheter was then advanced through the right heart to obtain right atrial pressure (RAP), RV pressure (systolic, diastolic), pulmonary artery pressure (PAP) (systolic, diastolic, mean) and pulmonary capillary wedge pressure (PCWP) in end-expiration. All measurements were derived from three consecutive beats, which were averaged for the final analysis. Cardiac output (CO) was measured using indirect Fick’s method [2] and transpulmonary gradient (TPG) calculated as the difference between mean PAP and PCWP. Pulmonary vascular resistance (PVR) was calculated as TPG divided by CO.[3] PH was defined and categorised according to current guidelines as follows: pre-capillary PH (mean PAP >20 mmHg, PCWP ≤15 mmHg, PVR ≥2 WU); isolated post-capillary PH (mean PAP >20 mmHg, PCWP >15 mmHg, PVR <2 WU); and combined pre- and post-capillary PH (mean PAP >20 mmHg, PCWP >15 mmHg, PVR >2 WU).[1]

**Bi-ventricular three-dimensional echocardiographic volume measurement**

All TTE images were acquired using an EPIQ CVx v7.0 system (Philips Healthcare, Netherlands) and X5-1c transducer. Full-volume 3D datasets of the LV and RV were acquired during a breath hold from apical 4-chamber and RV-focused views, respectively.[4] Images were then optimised to include the entire ventricular cavity within the highest possible frame rate scan volume. Volume measurements were performed semi-automatically using TomTec Arena (Tomtec Imaging Systems GmbH, Unterschleißheim, Germany). Ventricular endocardial borders were identified at end-diastole and end-systole (with manual adjustments if necessary) to calculate 3D EDV, ESV and EF. Datasets were acquired using a minimum 4-beat full-volume mode in sinus rhythm, and in single-beat high-volume rate mode averaged over three different datasets in atrial fibrillation (AF).

**Potential energy calculation**

PE was derived from the triangle bordered by the ESPVR, volume axis and PVL, and calculated as 0.5 x ESP^2^/Ees.[5,6] Total hemodynamic efficiency was calculated as SW/PVA x 100.[7]

**Supplementary Figure 1.** Additional representative examples of study participant pressure volume loops.

Representative bi-ventricular pressure-volume loops acquired from three study participants before and after transcatheter aortic valve implantation.

**Abbreviations.** TAVI: transcatheter aortic valve implantation.

**References**

1. Humbert M, Kovacs G, Hoeper MM, Badagliacca R, Berger RMF, Brida M, et al. 2022 ESC/ERS Guidelines for the diagnosis and treatment of pulmonary hypertension. Eur Heart J. 2022;43:3618–731. https://doi.org/10.1093/eurheartj/ehac237

2. Fagard R, Conway J. Measurement of cardiac output: Fick principle using catheterization. Eur Heart J. 1990;11 Suppl I:1–5. https://doi.org/10.1093/eurheartj/11.suppl_i.1

3. Eleid MF, Padang R, Pislaru SV, Greason KL, Crestanello J, Nkomo VT, et al. Effect of Transcatheter Aortic Valve Replacement on Right Ventricular-Pulmonary Artery Coupling. JACC Cardiovasc Interv. 2019;12:2145–54. https://doi.org/10.1016/j.jcin.2019.07.025

4. Lang RM, Badano LP, Tsang W, Adams DH, Agricola E, Buck T, et al. EAE/ASE recommendations for image acquisition and display using three-dimensional echocardiography. J Am Soc Echocardiogr. 2012;25:3–46. https://doi.org/10.1016/j.echo.2011.11.010

5. Reil J-C, Reil G-H, Hecker N, Sequeira V, Borer JS, Stierle U, et al. Reduced left ventricular contractility, increased diastolic operant stiffness and high energetic expenditure in patients with severe aortic regurgitation without indication for surgery. Interact Cardiovasc Thorac Surg. 2021;32:29–38. https://doi.org/10.1093/icvts/ivaa232

6. Suga H. Ventricular energetics. Physiol Rev. 1990;70:247–77. https://doi.org/10.1152/physrev.1990.70.2.247

7. Sunagawa K, Maughan WL, Burkhoff D, Sagawa K. Left ventricular interaction with arterial load studied in isolated canine ventricle. Am J Physiol. 1983;245:H773-780. https://doi.org/10.1152/ajpheart.1983.245.5.H773
